# Irisin Is Controlled by Farnesoid X Receptor and Regulates Cholesterol Homeostasis

**DOI:** 10.3389/fphar.2019.00548

**Published:** 2019-05-28

**Authors:** Hong Li, Jing Shen, Tong Wu, Jiangying Kuang, Qinhui Liu, Shihai Cheng, Shiyun Pu, Lei Chen, Rui Li, Yanping Li, Min Zou, Zhiyong Zhang, Wei Jiang, Aijuan Qu, Jinhan He

**Affiliations:** ^1^Department of Pharmacy, State Key Laboratory of Biotherapy, Collaborative Innovation Center of Biotherapy, West China Hospital, Sichuan University, Chengdu, China; ^2^Laboratory of Clinical Pharmacy and Adverse Drug Reaction, State Key Laboratory of Biotherapy, Collaborative Innovation Center of Biotherapy, West China Hospital, Sichuan University, Chengdu, China; ^3^State Key Laboratory of Biotherapy, Molecular Medicine Research Center – Collaborative Innovation Center of Biotherapy, West China Hospital, Sichuan University, Chengdu, China; ^4^Department of Physiology and Pathophysiology, School of Basic Medical Sciences, Capital Medical University, Beijing, China

**Keywords:** FNDC5/Irisin, FXR, hyperlipidemia, atherosclerosis, ApoE-/-

## Abstract

**Objective:**

The aim of this study was to investigate whether the nuclear receptor farnesoid X receptor (FXR) could regulate FNDC5/Irisin expression and the role of Irisin in hyperlipidemia and atherosclerosis in ApoE-/- mice.

**Methods and Results:**

We treated primary human hepatocytes, HepG2 cells, and Rhesus macaques with FXR agonist (CDCA, GW4064, and ivermectin). FNDC5 expression was highly induced by CDCA and GW4064 in hepatocytes, HepG2 cells, and the circulating level of Irisin increased in Rhesus macaques. Luciferase reporter and CHIP assays were used to determine whether FXR could regulate FNDC5 promoter activity. Irisin-ApoE-/- and ApoE-/- mice were used to study the metabolic function of Irisin in dyslipidemia and atherosclerosis. Irisin-ApoE-/- mice showed improved hyperlipidemia and alleviated atherosclerosis as compared with ApoE-/- mice. Irisin upregulated the expression of Abcg5/Abcg8 in liver and intestine, which increased the transport of biliary cholesterol and fecal cholesterol output.

**Conclusion:**

Activation of FXR induces FNDC5 mRNA expression in human and increased the circulating level of Irisin in Rhesus macaques. FNDC5/Irisin is a direct transcriptional target of FXR. Irisin may be a novel therapeutic strategy for dyslipidemia and atherosclerosis.

## Introduction

Cholesterol plays an important role in cellular membranous structures. Its metabolites, such as bile acids, oxysterols, certain vitamins, and steroid hormones, are essential for various cellular functions (Grebe and Latz, 2013). However, accumulation of excess cholesterol can be harmful, leading to a wide variety of cellular toxicities, and human diseases such as atherosclerosis (Spady, 1999). Cholesterol can be mobilized and excreted to prevent atherosclerosis (Fitzgerald et al., 2010). Cholesterol export is mediated by several membrane cholesterol transporters, including ATP-binding cassette (Abc) transporters ABCG1, and ABCG1. Abca1 plays a crucial role in the efflux of cellular cholesterol to APOA-I, whereas ABCG1 promotes cellular cholesterol export to high-density lipoprotein (HDL) (Ye et al., 2011).

Cholesterol homeostasis is maintained mainly by intestinal absorption, conversion into bile acids and biliary and fecal excretion. Intestinal cholesterol absorption is mediated by Niemann-Pick C1-Like 1 (Npc1l1) (Altmann et al., 2004). Cholesterol can be converted into bile acids in the liver via the classical pathway (mainly by Cyp7a1 and Cyp8b1) or alternative pathways (mainly by Cyp7b1 and Cyp27a1) (Pandak et al., 2002; Hebanowska, 2011). Cholesterol excretion from the body is principally mediated by the Abc transporters ABCG5 and ABCG8 (Berge et al., 2000). In the liver, ABCG5/ABCG8 promote efficient secretion of cholesterol into bile (Graf et al., 2003; Wang et al., 2015). In the intestine, ABCG5/ABCG8 contribute to cholesterol excretion by transporting cholesterol into gut lumen (Graf et al., 2003; Wang et al., 2015).

Irisin is a novel polypeptide hormone released into the circulation by proteolytics from fibronectin type III domain-containing protein 5 (FNDC5) (Bostrom et al., 2012), which is highly expressed in heart, liver, and skeletal muscle (Huh et al., 2012). Circulating Irisin is positively correlated with HDL-cholesterol level and negatively correlated with total cholesterol level in women with normal weight obesity (Mehrabian et al., 2015). Benton et al. (2010) showed a significant association between Irisin and HDL-cholesterol levels in healthy non-diabetic subjects. Thus, Irisin might have a protective role in cardiovascular disease.

Farnesoid X receptor (FXR) is a ligand-activated transcription factor and a nuclear hormone receptor that regulates multiple biological processes. FXR can be activated by chenodeoxycholic acid (CDCA), GW4064, and ivermectin (Jin et al., 2013; Xu et al., 2015). FXR controls the expression of multiple genes that are key to many aspects of metabolism. Activation of FXR increases reverse cholesterol transport (Jonker et al., 2012). Consistent with the role of FXR in reverse cholesterol transport, activation of FXR protects against the development of atherosclerosis (Xu et al., 2016). Although the function of FXR in cholesterol metabolism is widely known, whether FXR may affect cholesterol metabolism by regulating Irisin is unknown.

In this study, we showed that FNDC5/Irisin is regulated by FXR. Overexpression of Irisin alleviated atherosclerosis in ApoE-/- mice. Mechanistic studies revealed that Irisin enhanced cholesterol efflux from the body by hepatobiliary secretion via upregulating Abcg5/8 expression. Our results demonstrate a novel pathway of Irisin regulation and provide a potential therapeutic target for the treatment of atherosclerosis.

## Materials and Methods

### Cell Culture, Animal, Drug Treatment, and Diet

All cell lines were cultured under standard conditions at 37°C, 5% CO_2_ in a humidified incubator. HepG2 cells (ATCC HB8065) were maintained in Earle’s modified Eagle’s medium (EMEM) supplemented with 10% fetal bovine serum (FBS). HepG2 cells were seeded in a 6-well and treated with CDCA (100 μM) or GW4064 (2.5 μM) for 24 h and mRNAs were determined by qPCR.

Primary human hepatocytes were isolated from freshly excised human liver tissue from donors who underwent elective operations (e.g., hepatectomy) at the Department of Surgery, West China hospital, Sichuan University. All the donors gave their written informed consent for participation in the study. The study protocol was approved by the Research Ethics Committee of West China Hospital of Sichuan University (ChiCTR ECS 14004441). All the procedures followed the Declaration of Helsinki principles.

The Institutional Animal Care and Use Committee of Sichuan University, Chengdu, China, approved all studies. The investigation conforms to the Guide for the Care and Use of Laboratory Animals published by the United States National Institutes of Health. Rhesus macaques were housed in climate-controlled conditions with 12-h light/dark cycles. Monkeys were provided water *ad libitum* and fed twice a day with a normal diet (*n* = 8). For ivermectin treatment, monkeys received a single subcutaneous injection with ivermectin (0.4 mg/kg) and blood was collected 24 h later. ApoE-/- mice were purchased from the Jackson Laboratory (Bar Harbor, ME, United States) and were maintained on a C57BL/6J background. Irisin transgenic (Irisin-Tg) mice on a C57BL/6J background were created by Shanghai Biomodel Organism Science & Technology Development as we reported previously (Mo et al., 2016). Irisin-Tg mice were crossed with ApoE-/- mice to generate Irisin-ApoE-/- mice (*n*_WT_ = 34, *n*_Tg_ = 36, male). Mice were fed a chow diet or Western diet for 8 weeks (42% of kcal from fat, 0.2% cholesterol, Harland Tekland, TD88137). Mice were euthanized with CO_2_.

### Luciferase Reporter Gene Assay and Chromatin Immunoprecipitation (ChIP) Assay

The FNDC5 gene promoter (-3030 to -1890 bp) or mutant FNDC5 gene promoter was cloned into the pGL3-Basic vector. HEK293T cells were transfected with reporter constructs in 48-well plates as described (The viability of cells were above 90% under high magnification microscope). Cells transfected with plasmid and treated with FXR agonists, GW4064 or CDCA for 24 h, then collected and lysed, and luciferase activity was measured. ChIP assay was performed essentially as described previously (Mo et al., 2016; Kuang et al., 2017). Briefly, cells transfected with FXR plasmid and treated with or without FXR agonists (CDCA (100 μM) or GW4064 (2.5 μM)) for 24 h. ChIP assays according to the protocol of ChIP assay kit (MAGNA0002). After formaldehyde cross-linking, the protein-DNA complexes were obtained by Immunoprecipitated with antibody to FXR (sc-13063) or control Immunoglobulin G (IgG) and quantified by quantitative PCR (qPCR). PCR products were detected by 2% agarose gel electrophoresis.

### Evaluation of Atherosclerosis

For *en face* quantification of atherosclerotic lesions in the entire mouse aorta, whole aortas were collected, opened longitudinally, and stained with Oil-red O (*n*_WT_ = 10, *n*_TG_ = 10, *n*_WTD_-_WT_ = 12, *n*_WTD-TG_ = 12). Images of aortas were captured by microscopy (Nikon, Tokyo, Japan), analyzed with Image J software, and presented as the percentage of lesion area in the whole aorta. For quantification of atherosclerotic lesions in the aortic sinus, serial cryosections (6 μm) were stained with Oil-red O, then counterstained with hematoxylin. Paraffin sections (4 μm) were stained with hematoxylin and eosin.

### Blood Cholesterol, Triglycerides, and Irisin Assessment

Serum and biliary levels of cholesterol and triglycerides were measured by using kits (Biosino, Beijing). To measure hepatic and fecal levels of triglycerides and cholesterol, hepatic and fecal lipid contents were extracted as described (Reza et al., 2017) and measured by using commercial assay kits. Serum Irisin level in blood samples was measured by commercially available enzyme-linked immunosorbent assay (ELISA) kits (Phoenix Pharmaceuticals, Cat EK-067-29).

### Quantitative Real-Time RT-PCR

For real-time PCR analysis, total hepatic and intestinal RNA was extracted with Trizol reagent (Invitrogen, Carlsbad, CA, United States) and reverse-transcribed into cDNA by reverse transcription with Superscript RT III enzyme (TaKaRa, Kyoto, Japan). Quantitative real-time PCR involved use of the CFX96 PCR system (Bio-Rad, CA, United States). The primers for RT -PCR used in the studies are provided in Supplementary Table 1.

### Western Blot Analysis

Liver and intestines were lysed by using lysis buffer and underwent western blot analysis with the primary antibodies anti-Abcg5 (1:1000; sc-25796, Santa Cruz, CA, United States), anti-Abcg8 (1:1000; sc-30111, Santa Cruz, CA, United States), and β-tubulin (1:1000; 200608, Zen Bio Science, China). Bands were visualized by using the LI-COR (Lincoln, NE, United States) Odyssey System. Quantification of band intensity involved using Image Studio v4.0 (LI-COR).

### Statistical Analysis

Data are expressed as mean ± SEM. Statistical significance was determined by Student’s *t* test (unpaired two-tailed) or one-way ANOVA (Tukey’s test) for multiple comparisons. *P* < 0.05 was considered statistically significant.

## Results

### FNDC5 Is Regulated by FXR

**FIGURE 1:**
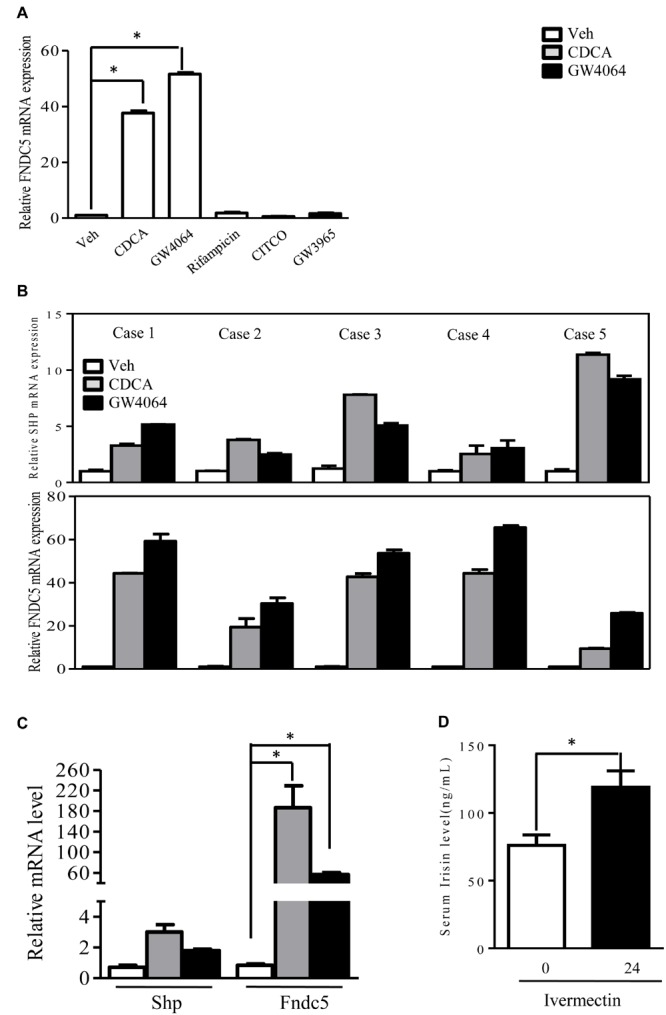
Activation of farnesoid X receptor (FXR) regulates fibronectin type III domain-containing protein 5 (FNDC5) expression. **(A)** Cultured human hepatocytes were treated with different nuclear receptor agonists: CDCA (100 μM) and GW4064 (2.5 μM), agonists for FXR; Rif (5 μM), an agonist for human PXR; CITCO (100 nM), an agonist for human CAR; and GW (10 μM), an agonist for liver X receptor α (LXRα) (*n* = 3) for 24 h. **(B)** FXR agonist induced (small heterodimer partner) SHP and fibronectin type III domain-containing protein 5 (FNDC5) mRNA expression in cultured human hepatocytes from 5 different cases. **(C)** HepG2 cells were treated with CDCA (100 μM) or GW4064 (2.5 μM) for 24 h (*n* = 4). **(D)** Rhesus macaques were subcutaneously injected with single dose of ivermectin (0.4 mg/kg) and blood was collected 24 h later (*n* = 8). CDCA, chenodeoxycholic acid; Rif, rifamycin; GW, GW3965. Data are mean ± SEM (*n* = 4). ^∗^*P* < 0.05.

To investigate the regulation of FNDC5/Irisin, we treated primary human hepatocytes with different nuclear receptors agonist (CITCO for CAR, CDCA/GW4064 for FXR, Rif for PXR, GW3965 for LXR). FNDC5 expression was highly induced by CDCA and GW4064, natural, and synthetic ligands for FXR, respectively. This regulation appeared to be FXR-specific because activation of pregnane X receptor or liver X receptor had no effect on FNDC5 expression (Figure 1A). To further confirm this result, we treated primary human hepatocytes from 5 different cases with CDCA and GW4064 for 24 h. As expected, CDCA or GW4064 increased the mRNA expression of small heterodimer partner (SHP), an FXR target gene. In all 5 cases, the expression of FNDC5 was highly induced by both CDCA and GW4064 (Figure 1B). A hepatocarcinoma cell line, HepG2 (ATCC HB-8065), also showed regulation of FNDC5 by FXR (Figure 1C). To investigate whether FXR could regulate FNDC5 expression *in vivo*, Rhesus macaques were subcutaneously injected with a single dose of ivermectin, a novel ligand for FXR^15^. Serum Irisin level was significantly increased 24 h after ivermectin injection (Figure 1D). Hence, FXR regulated FNDC5 expression both *in vitro* and *in vivo*. All data are mean ± SEM, ^∗^*p* < 0.05.

### FNDC5 Is a Direct Transcriptional Target of FXR

**FIGURE 2:**
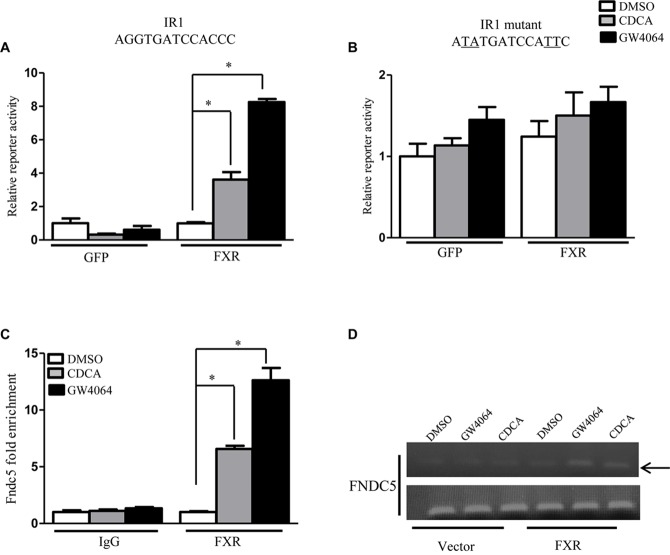
Fibronectin type III domain-containing protein 5 (FNDC5) is transcriptional target gene of FXR. **(A,B)** Luciferase assay of transient transfection in HEK293T cells with the FNDC5 natural and mutant promoter reporter (*n* = 3). **(C)** HepG2 cells were transfected with or without FXR vector, then treated with CDCA (100 μM) or GW4064 (2.5 μM) for 24 h. ChIP was performed with anti-FXR antibody (*n* = 3). **(D)** The relative band intensity of PCR products revealed the recruitment of FXR to FNDC5. Data are mean ± SEM. ^∗^*P* < 0.05.

Having demonstrated that FXR could regulate FNDC5 expression both *in vitro* and *in vivo*, we further investigated whether FNDC5 is a direct transcriptional target of FXR. Examination of the FNDC5 promoter revealed a putative inverted repeat by one nucleotide (IR-1) binding site located at -2619 bp (Supplementary Figure 1). Luciferase reporter assays were used to determine whether FXR could regulate FNDC5 promoter activity with a reporter gene that contains the natural promoter of FNDC5 (-1890 to -3030 bp). On transient transfection assay of HepG2 cells, reporter activity was induced about 4- and 8-fold in the presence of CDCA and GW4604 (ligands of FXR), respectively (Figure 2A). Mutation of the IR-1 binding site resulted in loss of the FXR effect on reporter activity (Figure 2B). ChIP assay was used to measure FXR transcriptional activity on the FNDC5 gene promoter. Consistent with the luciferase assay, treatment with CDCA/GW4064 significantly increased the recruitment of FXR to the FNDC5 promoter (Figure 2C,D). Thus, Irisin is a direct transcriptional target of FXR. All data are mean ± SEM, ^∗^*p* < 0.05.

### Improved Lipid Profiles in Irisin-ApoE-/- Mice

**FIGURE 3:**
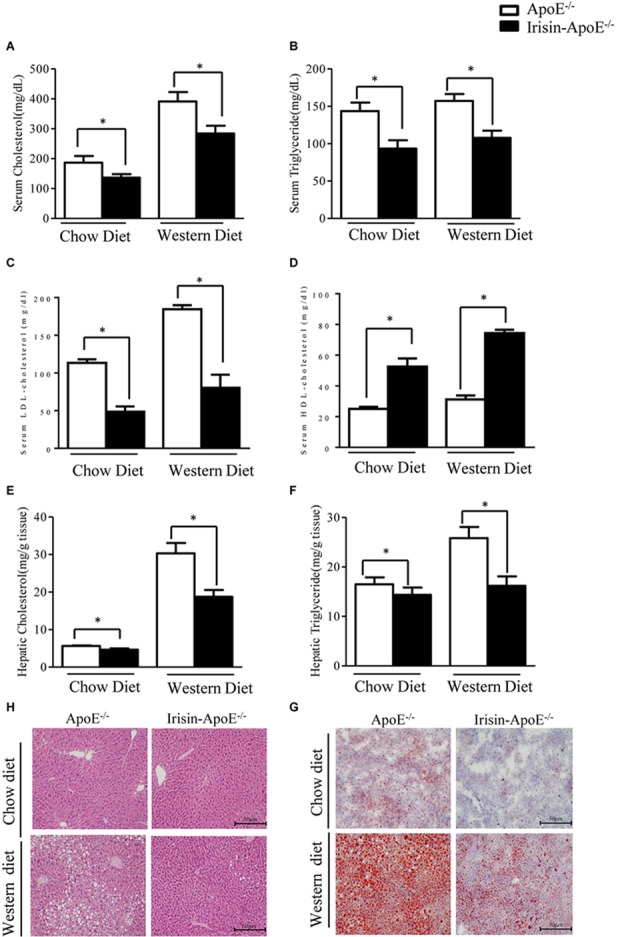
Serum parameters and hepatic lipid in ApoE-/- and Irisin-ApoE-/- mice. ApoE-/- and Irisin-ApoE-/- mice 20 weeks old were fed a chow or Western diet for 8 weeks. **(A–D)** Serum levels of cholesterol (*n* = 7), triglycerides (*n* = 7), LDL-cholesterol, and HDL-cholesterol (*n* = 4) in mice. **(E,F)** Hepatic cholesterol and triglycerides levels in mice (*n* = 6). **(G,H)** Representative H&E and Oil-red O staining of liver sections. Data are mean ± SEM. ^∗^*P* < 0.05.

For the limitations of the experimental conditions, there were none human or Rhesus macaques used to study the metabolic function of Irisin. However, the protein sequence is highly conserved (Jedrychowski et al., 2015). The human and murine sequences are identical. To understand the potential effect of Irisin on cholesterol metabolism, a function of FXR, Irisin-tg mice were bred with ApoE-/- mice (Mo et al., 2016). We then evaluated the effect of Irisin on hyperlipidemia in ApoE-/- mice fed a chow or Western diet. Serum levels of cholesterol and triglycerides were significantly lower in Irisin-ApoE-/- than ApoE-/- mice (Figure 3A,B) under both chow and Western diets. The low level of cholesterol was mainly due to a decrease in low-density lipoprotein (LDL)-cholesterol level (Figure 3C). HDL-cholesterol level was increased in Irisin-ApoE-/- mice (Figure 3D). Hepatic cholesterol and triglycerides levels were also reduced in Irisin-ApoE-/- mice (Figure 3E,F). In agreement, Oil-red O and H&E staining further confirm the improved hepatic lipid profile in Irisin-ApoE-/- mice (Figure 3G,H). All data are mean ± SEM, ^∗^*p* < 0.05.

### Irisin Promoted Cholesterol Transport in Liver

**FIGURE 4:**
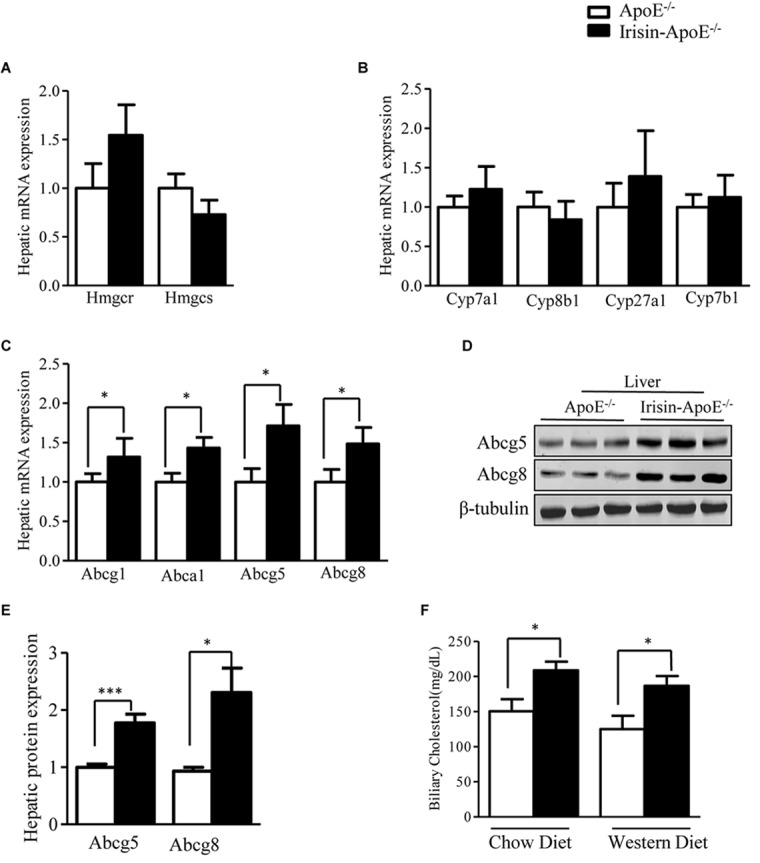
Irisin increases hepatic expression of Abcg5 and Abcg8 in ApoE-/- mice. **(A,B)** Real-time PCR analysis of hepatic mRNA expression of cholesterol and bile acid synthesis genes (*n* = 8). **(C)** mRNA expression of hepatic cholesterol transporters (*n* = 6). **(D,E)** Protein levels of hepatic Abcg5 and Abcg8 in mice (*n* = 3). **(F)** Biliary cholesterol level in mice (*n* = 5). Data are means ± SEM. ^∗^*P* < 0.05; ^∗∗∗^*P* < 0.001.

To gain insights in the mechanisms by which Irisin prevented hyperlipidemia in ApoE-/-mice, we analyzed the expression of genes involved in cholesterol metabolism. First, we evaluated the expression of genes involved in cholesterol synthesis. The hepatic mRNA levels of Hmgcr and Hmgcs were not changed among the genotypes (Figure 4A), nor was expression of genes that convert cholesterol into bile acid, such as Cyp7a1, Cyp8b1, Cyp27a1, and Cyp7b1 (Figure 4B). The efflux of cholesterol from hepatocytes is mediated by two pathways. On the sinusoidal side, cholesterol is transported to HDL via Abca1 and Abcg1 (Ye et al., 2011). On the apical side, cholesterol is transported into bile via Abcg5 and Abcg8 (Graf et al., 2003). The hepatic mRNA levels of Abca1 and Abcg1 were significantly higher in Irisin-ApoE-/- than ApoE-/- mice (Figure 4C). The increased expression of Abca1 and Abcg1 may explain the higher serum level of HDL-cholesterol. Moreover, overexpression of Irisin significantly increased both the mRNA and protein expression of Abcg5 and Abcg8 (Figure 4C–E). We also detect the mRNA level in Irisin-tg and WT mice. The mRNA level of Abcg5 and Abcg8 were significantly increased in Irisin-tg mice compare to WT mice (data not shown). As a result, the biliary cholesterol level was higher in Irisin-ApoE-/- mice (Figure 4F). All data are mean ± SEM, ^∗^*p* < 0.05.

### Irisin Increases the Intestinal Expression of Abcg5 and Abcg8

**FIGURE 5:**
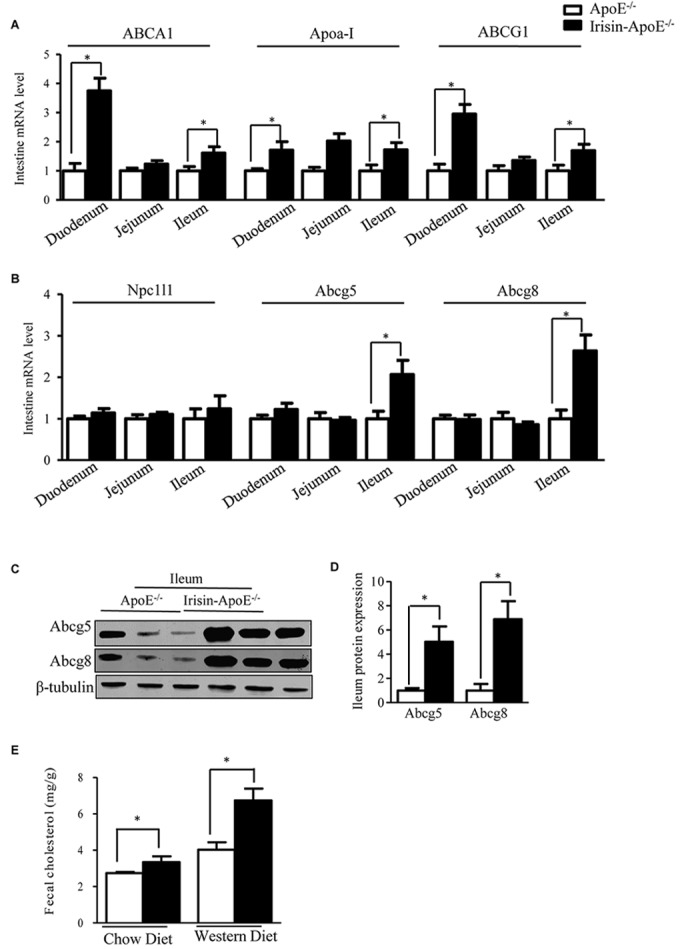
Intestinal Abcg5 and Abcg8 levels were increased in Irisin-ApoE-/- mice. **(A,B)** mRNA expression of cholesterol transporters in duodenum, jejunum, and ileum of mice (*n* = 6). **(C,D)** Protein expression of Abcg5 and Abcg8 in the ileum (*n* = 3) and **(E)** fecal cholesterol output in mice (*n*_WT_ = 6, *n*_TG_ = 4, *n*_WTD-WT_ = 6, *n*_WTD-TG_ = 6). Data are mean ± SEM. ^∗^*P* < 0.05.

Intestine is also crucially involved in the maintenance of serum HDL levels (Brunham et al., 2006). Intestinal Abca1 directly contributes to HDL biogenesis (Gelissen et al., 2006). Abcg1 has been shown to facilitate cholesterol efflux from cells to HDL particles and is proposed to participate in the generation of HDL particles in concert with Abca1 (Gelissen et al., 2006). Irisin increased the intestinal expression of Abca1 (Figure 5A). ABCG5 and ABCG8 are also expressed in the intestine and are involved in intestinal cholesterol absorption. The mRNA and protein expression of Abcg5 and Abcg8 was increased in ileum (Figure 5B–D). We also detect the mRNA level in Irisin-tg and WT mice. The mRNA level of Abcg5 and Abcg8 were significantly increased in Irisin-tg mice compare to WT mice (data not shown). Of note, the Npc1l1 level was not changed in Irisin-tg mice (Figure 5B). Moreover, fecal cholesterol level was higher in Irisin-ApoE-/- than ApoE-/- mice (Figure 5E). These studies indicate that overexpression of Irisin increased the intestinal expression of Abcg5/Abcg8 and promoted cholesterol excretion into feces. All data are mean ± SEM, ^∗^*p* < 0.05.

### Irisin Alleviated Atherosclerotic Plaque Formation in ApoE-/- Mice

**FIGURE 6:**
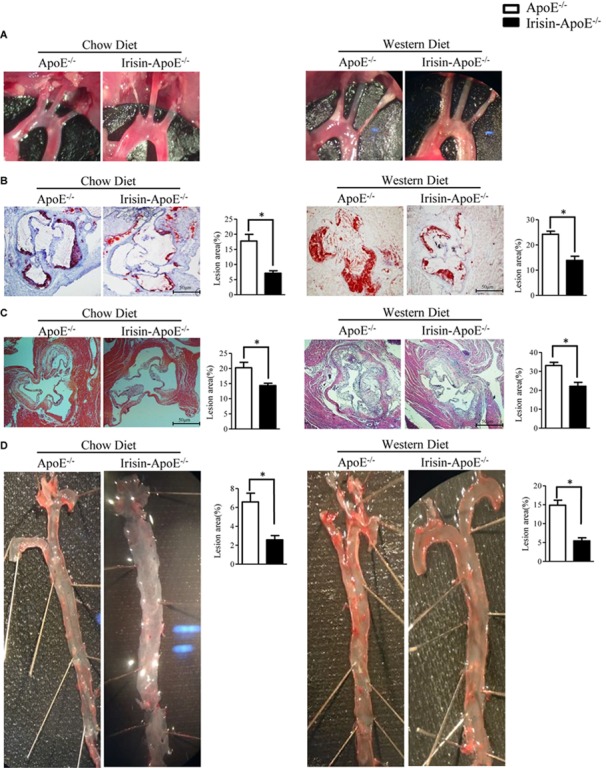
Irisin alleviated atherosclerotic plaque formation in ApoE-/- mice. **(A)** Aortic arch in ApoE-/- and Irisin-ApoE-/- mice. **(B,C)** Oil-red O and H&E (*n*_WT_ = 5, *n*_TG_ = 6) staining of aortic root. **(D)** Oil-red O staining of whole aortas of ApoE-/- and Irisin-ApoE-/- mice (*n*_WT_ = 5, *n*_TG_ = 6). Data are mean ± SEM. ^∗^*P* < 0.05.

Because LDL-cholesterol level was decreased and HDL-cholesterol level increased in Irisin-tg mice, we hypothesized that Irisin may play an important role in atherosclerosis. To investigate the effect of Irisin on atherosclerosis, Irisin-ApoE-/- and ApoE-/- mice were fed a chow or Western diet for 2 months. Analysis of plaque in the aortic arches demonstrated significantly less atherosclerotic plaques in Irisin -ApoE-/- than ApoE-/- mice and also less lipid at the cardiac/aortic junction (Figure 6A,B). Furthermore, the total lesion area at the cardiac/aortic junctions was significantly decreased in Irisin-ApoE-/- than ApoE-/- mice fed a chow or Western diet (Figure 6C). The whole aorta showed 50–70% lower lipid staining in Irisin-ApoE-/- than ApoE-/- mice fed a chow or Western diet (Figure 6D). These studies indicate that Irisin alleviated atherosclerotic plaque formation, most likely representing atherosclerotic severity in the ApoE-/- mice model. All data are mean ± SEM, ^∗^*p* < 0.05.

## Discussion

Here we show that activation of FXR induced FNDC5 mRNA level in the human hepatocytes and increased circulating Irisin level in rhesus monkeys. We also showed that FNDC5 is a direct transcriptional target of FXR. *In vivo*, overexpression of Irisin alleviated atherosclerosis in ApoE-/- mice. Mechanistic studies revealed that Irisin enhanced cholesterol efflux from the body by upregulating hepatic and intestinal Abcg5/Abcg8 expression.

In our previous study, we showed that FNDC5/Irisin is regulated by CAR as a direct target gene in mice (Mo et al., 2016). However, in the present study, FNDC5/Irisin was controlled by FXR in primates. This species difference is likely due to unconcerned sequences located in the promotor region of FNDC5. We found the classical nuclear receptor-response element of FXR, IR-1, located in the promoter of FNDC5 in primates, whereas a nuclear receptor-response element of CAR, the direct repeat spaced by five nucleotides (DR5-type), was located in the promoter of FNDC5 in rodent. This could explain why FNSC5 is differentially regulated in primates and rodents.

Irisin has been reported to be associated with cholesterol metabolism (Tang et al., 2015). Circulating levels of Irisin were positively correlated with HDL-cholesterol but negatively with total cholesterol, LDL-cholesterol, and triglycerides levels (Huh et al., 2012; Tang et al., 2015; Dozio et al., 2017). Lentivirus-mediated overexpression of FNDC5/Irisin or subcutaneous perfusion of Irisin reduced blood levels of triglycerides, cholesterol, free fatty acid, and glucose in obese mice (Xiong et al., 2015). In addition, adenovirus-overexpressed Irisin and Irisin-tg mice are protected against high-fat-diet–induced hepatic steatosis and suppressed triglycerides accumulation via the AMPK pathway (Mo et al., 2016). Subcutaneous infusion of Irisin for 2 weeks reduced plasma and hepatic cholesterol levels in obese mice induced by an high fat diet via AMPK signaling (Tang et al., 2016).

We found that Irisin decreased blood cholesterol level. The lower level of cholesterol seen in Irisin-transgenic than wild-type mice was mainly due to a decrease in level of LDL-cholesterol but not HDL-cholesterol. Cholesterol homeostasis in the body is maintained mainly by *de novo* synthesis, intestinal absorption, and biliary and fecal excretion (Litvinov et al., 2016). The *de novo* synthesis of cholesterol seems not to be affected by Irisin because the hepatic levels of Hmgcr and Hmgcs were not changed in Irisin-ApoE-/- mice. The intestinal cholesterol absorption is determined by Npc1L1 and ABCG5/ABCG8. Npc1l1 promoted cholesterol absorption by mediating cholesterol transport across the brush border membrane (Hui et al., 2008). However, Abcg5 and Abcg8 form an obligate heterodimer that limits intestinal absorption (Yu et al., 2014). We found that Irisin increased the intestinal expression of Abcg5 and Abcg8 but did not change that of Npc1l1. The increased expression of Abcg5/Abcg8 may decrease the intestinal absorption of cholesterol. Hepatic ABCG5/ABCG8 plays a key role in biliary secretion of cholesterol. We found that overexpression of Irisin increased the mRNA and protein levels of Abcg5/Abcg8 in liver. Consistent with the higher expression of Abcg5/Abcg8, the biliary concentration of cholesterol was higher. Therefore, the higher expression of Abcg5/Abcg8 in both liver and intestine might work together to prevent excess cholesterol accumulation in the body.

Irisin has been found as an anti-atherogenic transcription factor (Lu et al., 2015; Zhang et al., 2016). First, Lu et al. (2015) found that tail vein injection of Irisin protected against atherosclerosis in diabetic ApoE-/- mice by ameliorating high glucose-induced endothelial dysfunction and apoptosis. Recently, Zhang et al. (2016) demonstrated that intraperitoneal injection of Irisin decreased inflammation and cell apoptosis in aortic tissues. We found a new role of Irisin in cholesterol homeostasis in Irisin-transgenic mice, an animal model to expression Irisin a long-term.

## Conclusion

We reveal a novel pathway regulating expression of Irisin. Overexpression of Irisin decreased hyperlipidemia and alleviated atherosclerosis in ApoE-/- mice. Irisin upregulated hepatic and intestinal Abcg5/Abcg8 expression, leading to increased biliary cholesterol excretion and fecal cholesterol output. Irisin may be a novel therapeutic strategy for dyslipidemia and atherosclerosis.

## Data Availability

The raw data supporting the conclusions of this manuscript will be made available by the authors, without undue reservation, to any qualified researcher.

## Ethics Statement

All the donors gave their written informed consent for participation in the study. The study protocol was approved by the Research Ethics Committee of West China Hospital of Sichuan University (ChiCTR ECS 15005551). All the procedures followed the Declaration of Helsinki principles.

## Author Contributions

HL designed and performed the experiments and wrote the manuscript. JS, TW, JK, QL, SC, SP, LC, RL, YL, MZ, and WJ helped with experiments. WJ, ZZ, and MZ contributed to the discussion and review of the manuscript. JH obtained the funding, designed the experiments, and wrote the manuscript. HL and JH were the guarantors of this work and, as such, had full access to all the data in the study and took responsibility for the integrity of the data and the accuracy of the data analysis. AQ contributed to the discussion and review of the manuscript.

## Conflict of Interest Statement

The authors declare that the research was conducted in the absence of any commercial or financial relationships that could be construed as a potential conflict of interest.

## Abbreviations

Abcg5/8: ATP-binding cassette transporters5/8
ApoE-/-: ApoE knockout
CAR: constitutive androstane receptor
CDCA: chenodeoxycholic acid
FNDC5: fibronectin type III domain-containing protein 5
FXR: farnesoid X receptor
GW: GW3965
Irisin-TG: irisin transgenic mice
LXR: liver X receptor
Rif: rifamycin

## References

[B1] Altmann S. W. Davis H. R. Jr. Zhu L. J. Yao X. Hoos L. M. Tetzloff G. (2004). Niemann-Pick C1 Like 1 protein is critical for intestinal cholesterol absorption. *Science* 303 1201–1204. 10.1126/science.1093131 14976318

[B2] Benton C. R. Holloway G. P. Han X. X. Yoshida Y. Snook L. A. Lally J. (2010). Increased levels of peroxisome proliferator-activated receptor gamma, coactivator 1 alpha (PGC-1alpha) improve lipid utilisation, insulin signalling and glucose transport in skeletal muscle of lean and insulin-resistant obese Zucker rats. *Diabetologia* 53 2008–2019. 10.1007/s00125-010-1773-1 20490453

[B3] Berge K. E. Tian H. Graf G. A. Yu L. Grishin N. V. Schultz J. (2000). Accumulation of dietary cholesterol in sitosterolemia caused by mutations in adjacent ABC transporters. *Science* 290 1771–1775. 10.1126/science.290.5497.1771 11099417

[B4] Bostrom P. Wu J. Jedrychowski M. P. Korde A. Ye L. Lo J. C. (2012). A PGC1-alpha-dependent myokine that drives brown-fat-like development of white fat and thermogenesis. *Nature* 481 463–468. 10.1038/nature10777 22237023 PMC3522098

[B5] Brunham L. R. Kruit J. K. Iqbal J. Fievet C. Timmins J. M. Pape T. D. (2006). Intestinal ABCA1 directly contributes to HDL biogenesis in vivo. *J. Clin. Invest.* 116 1052–1062. 10.1172/jci27352 16543947 PMC1401485

[B6] Dozio E. Passeri E. Cardani R. Benedini S. Aresta C. Valaperta R. (2017). Circulating irisin is reduced in male patients with type 1 and type 2 myotonic dystrophies. *Front. Endocrinol.* 8:320. 10.3389/fendo.2017.00320 29184538 PMC5694592

[B7] Fitzgerald M. L. Mujawar Z. Tamehiro N. (2010). ABC transporters, atherosclerosis and inflammation. *Atherosclerosis* 211 361–370. 10.1016/j.atherosclerosis.2010.01.011 20138281 PMC2888932

[B8] Gelissen I. C. Harris M. Rye K. A. Quinn C. Brown A. J. Kockx M. (2006). ABCA1 and ABCG1 synergize to mediate cholesterol export to apoA-I. *Arterioscler. Thromb. Vasc. Biol.* 26 534–540. 10.1161/01.atv.0000200082.58536.e1 16357317

[B9] Graf G. A. Yu L. Li W. P. Gerard R. Tuma P. L. Cohen J. C. (2003). ABCG5 and ABCG8 are obligate heterodimers for protein trafficking and biliary cholesterol excretion. *J. Biol. Chem.* 278 48275–48282. 10.1074/jbc.m310223200 14504269

[B10] Grebe A. Latz E. (2013). Cholesterol crystals and inflammation. *Curr. Rheumatol. Rep.* 15:313. 10.1007/s11926-012-0313-z 23412688 PMC3623938

[B11] Hebanowska A. (2011). Mechanisms of bile acid biosynthesis regulation–autoregulation by bile acids. *Postepy Biochem.* 57 314–323. 22235657

[B12] Huh J. Y. Panagiotou G. Mougios V. Brinkoetter M. Vamvini M. T. Schneider B. E. (2012). FNDC5 and irisin in humans: I. predictors of circulating concentrations in serum and plasma and II. mRNA expression and circulating concentrations in response to weight loss and exercise. *Metab. Clin. Exp.* 61 1725–1738. 10.1016/j.metabol.2012.09.002 23018146 PMC3614417

[B13] Hui D. Y. Labonte E. D. Howles P. N. (2008). Development and physiological regulation of intestinal lipid absorption. III. intestinal transporters and cholesterol absorption. *Am. J. Physiol. Gastrointest. Liver Physiol.* 294 G839–G843. 10.1152/ajpgi.00061.2008 18276831

[B14] Jedrychowski M. P. Wrann C. D. Paulo J. A. Gerber K. K. Szpyt J. Robinson M. M. (2015). Detection and quantitation of circulating human irisin by tandem mass spectrometry. *Cell Metab.* 22 734–740. 10.1016/j.cmet.2015.08.001 26278051 PMC4802359

[B15] Jin L. Feng X. Rong H. Pan Z. Inaba Y. Qiu L. (2013). The antiparasitic drug ivermectin is a novel FXR ligand that regulates metabolism. *Nat. Commun.* 4:1937. 10.1038/ncomms2924 23728580

[B16] Jonker J. W. Liddle C. Downes M. (2012). FXR and PXR: potential therapeutic targets in cholestasis. *J. Ster. Biochem. Mol. Biol.* 130 147–158. 10.1016/j.jsbmb.2011.06.012 21801835 PMC4750880

[B17] Kuang J. Zhang Y. Liu Q. Shen J. Pu S. Cheng S. (2017). Fat-specific sirt6 ablation sensitizes mice to high-fat diet-induced obesity and insulin resistance by inhibiting lipolysis. *Diabetes* 66 1159–1171. 10.2337/db16-1225 28250020

[B18] Litvinov D. F. Savushkin E. V. Garaeva E. A. Dergunov A. D. (2016). Cholesterol efflux and reverse cholesterol transport: experimental approaches. *Curr. Med. Chem.* 23 3883–3908. 10.2174/0929867323666160809093009 27516200

[B19] Lu J. Xiang G. Liu M. Mei W. Xiang L. Dong J. (2015). Irisin protects against endothelial injury and ameliorates atherosclerosis in apolipoprotein E-Null diabetic mice. *Atherosclerosis* 243 438–448. 10.1016/j.atherosclerosis.2015.10.020 26520898

[B20] Mehrabian S. Taheri E. Karkhaneh M. Qorbani M. Hosseini S. (2015). Association of circulating irisin levels with normal weight obesity, glycemic and lipid profile. *J. Diabetes Metab. Disord.* 15:17. 10.1186/s40200-016-0239-5 27354972 PMC4924282

[B21] Mo L. Shen J. Liu Q. Zhang Y. Kuang J. Pu S. (2016). Irisin is regulated by car in liver and is a mediator of hepatic glucose and lipid metabolism. *Mol. Endocrinol.* 30 533–542. 10.1210/me.2015-1292 27007446 PMC5414639

[B22] Pandak W. M. Ren S. Marques D. Hall E. Redford K. Mallonee D. (2002). Transport of cholesterol into mitochondria is rate-limiting for bile acid synthesis via the alternative pathway in primary rat hepatocytes. *J. Biol. Chem.* 277 48158–48164. 10.1074/jbc.m205244200 12368294

[B23] Reza M. M. Subramaniyam N. Sim C. M. Ge X. Sathiakumar D. McFarlane C. (2017). Irisin is a pro-myogenic factor that induces skeletal muscle hypertrophy and rescues denervation-induced atrophy. *Nat. Commun.* 8:1104. 10.1038/s41467-017-01131-0 29062100 PMC5653663

[B24] Spady D. K. (1999). Reverse cholesterol transport and atherosclerosis regression. *Circulation* 100 576–578. 10.1161/01.cir.100.6.576 10441091

[B25] Tang H. Yu R. Liu S. Huwatibieke B. Li Z. Zhang W. (2016). Irisin inhibits hepatic cholesterol synthesis via AMPK-SREBP2 signaling. *EBioMedicine* 6 139–148. 10.1016/j.ebiom.2016.02.041 27211556 PMC4856751

[B26] Tang S. Zhang R. Jiang F. Wang J. Chen M. Peng D. (2015). Circulating irisin levels are associated with lipid and uric acid metabolism in a Chinese population. *Clin. Exp. Pharmacol. Physiol.* 42 896–901. 10.1111/1440-1681.12439 26111934

[B27] Wang J. Mitsche M. A. Lutjohann D. Cohen J. C. Xie X. S. Hobbs H. H. (2015). Relative roles of ABCG5/ABCG8 in liver and intestine. *J. Lipid Res.* 56 319–330. 10.1194/jlr.M054544 25378657 PMC4306686

[B28] Xiong X. Q. Chen D. Sun H. J. Ding L. Wang J. J. Chen Q. (2015). FNDC5 overexpression and irisin ameliorate glucose/lipid metabolic derangements and enhance lipolysis in obesity. *Biochim. Biophys. Acta* 1852 1867–1875. 10.1016/j.bbadis.2015.06.017 26111885

[B29] Xu X. Xu X. Liu P. Zhu Z. Y. Chen J. Fu H. A. (2015). Structural basis for small molecule NDB (N-Benzyl-N-(3-(tert-butyl)-4-hydroxyphenyl)-2,6-dichloro-4-(dimethylamino) Benzamide) as a selective antagonist of farnesoid X receptor ( (FXR() in stabilizing the homodimerization of the receptor. *J. Biol. Chem.* 290 19888–19899. 10.1074/jbc.M114.630475 26100621 PMC4528148

[B30] Xu Y. Li F. Zalzala M. Xu J. Gonzalez F. J. Adorini L. (2016). Farnesoid X receptor activation increases reverse cholesterol transport by modulating bile acid composition and cholesterol absorption in mice. *Hepatology* 64 1072–1085. 10.1002/hep.28712 27359351 PMC5033696

[B31] Ye D. Lammers B. Zhao Y. Meurs I. Van Berkel T. J. Van Eck M. (2011). ATP-binding cassette transporters A1 and G1, HDL metabolism, cholesterol efflux, and inflammation: important targets for the treatment of atherosclerosis. *Curr. Drug Targets* 12 647–660. 10.2174/138945011795378522 21039336

[B32] Yu X. H. Qian K. Jiang N. Zheng X. L. Cayabyab F. S. Tang C. K. (2014). ABCG5/ABCG8 in cholesterol excretion and atherosclerosis. *Clin. Chim. Acta* 428 82–88. 10.1016/j.cca.2013.11.010 24252657

[B33] Zhang Y. Mu Q. Zhou Z. Song H. Zhang Y. Wu F. (2016). Protective effect of irisin on atherosclerosis via suppressing oxidized low density lipoprotein induced vascular inflammation and endothelial dysfunction. *PLoS One* 11:e0158038. 10.1371/journal.pone.0158038 27355581 PMC4927070

