# Supplementary material for: Irisin Is Controlled by Farnesoid X Receptor and Regulates Cholesterol Homeostasis
Source: Front Pharmacol. 2019 May 28;10:548. doi: 10.3389/fphar.2019.00548 (PMC6546903; doi:10.3389/fphar.2019.00548)
Supplement: Supplementary file 1 [file Image_1.pdf]

supplementary Fig.1

The IR-1 promoter sequence within the FNDC5 gene

5’·GGCTATAAAAAGGAAACTTCCTATTCTAAATGGGTACCCGGGGCTGGCTTGGGCACCTGCAGCCTG
AGTTAGGTGCTGAGACTCTGGACACGCAGGAGGGTCCAAATGCTGGTGACCCCATTAGGTGTCAATGT
GTAAGTGACCGGCCAGCCCTCAAGGGGGTCTCTGTGGAGTCCTCCAAATGAAGCCCAGTGAGTGCAGC
TTGGGAGGACAGAGGCGCACCCTTAGTCCCTACCCAGGTCCTCATGGTTTCACTCATTCTTTCACTGCT
GTGTTTCCAGAATATTACACAGGGCCTGGTCCATAATAGGTGCTCAATAAACATATGATGGAAGAATG
ATTTTTTTTCCATACAGTCAACAATCATTTAATGAGCCCCAACTGCATCGTAAGCCTTGCGCTAAGGGA
TGGGGTACATAAAGTGGCTGGGTTTTTTTGAGATGGAGTCTTTCTCTGTCACCCAGGCTGGAATGCAGT
GGTGTGCTCAGCTCACTGAAACCTCTGCATCCCAGGTTCAAATGATTCTCATGCCTCAGCCTCCAGAGT
AGCTGGGATTACAGGCACACACCACCATGCCCGGCTAATTTTGTATTTTTAGTAGAGACGGGGTTTCA
CCATGTTGGCCAGGCTGGTCTCGAACTCCTGACCTCAGGTGATCCACCCACCTCGGACTCCCACAGTGC
TGGGATTACAGGCGTGAGCCAGCGCCCTGGCCTGCAGTGGCTGCTTCTGACAGCTCCCTCCCAAGTTC
ACCCAGGGCTCCACTAAATCCTCCCAGCCAGCTCTCACTGGCTGCCAGATTCCAGGCACCAGGGCTTT
CCCCAGAATTAAGGCCCAAGCACCCCATGCATCATGAGGACTGAGAAGGCTGTACCCCCACAGTCAC
AACTCTCCAAATCCTCCAAGCCCCCTCCTTCAGCCGCCTTCTCTCTCAAGCCCTCAGGCACTGCCATCT
CCTCCCTCCCAATGCCCTACATCTATCTAGGTTTTACCCAAAGAGGCCAGCAGATGGCGCCAAGGTTCC
CCTTTCCCCAGCGCTGGACTGGAGGCCAAGACCCCACCAGGACCCTCCCTCCTTTTCTCCCCTGCAGGG
ACGGGAGTAGATTCTGTGTCCTCTAACTGAACCCTCTCCTTCCTGCTGGTTCCT-3’
