# Supplementary material for: Irisin Is Controlled by Farnesoid X Receptor and Regulates Cholesterol Homeostasis
Source: Front Pharmacol. 2019 May 28;10:548. doi: 10.3389/fphar.2019.00548 (PMC6546903; doi:10.3389/fphar.2019.00548)
Supplement: Supplementary file 2 [file Table_1.pdf]

supplementary Table 1

Table 1 The primers for RT -PCR

Genes	Sense	anti-sense
Cyp2b10	5'-TCGTTGAGCCAACCTTCA-3'	5'-ACTCCTCTTTCCCATCCC-3'
FNDC5	5'-AAGCACAAGGACTGACTCAAGC-3'	5'-CATGTCCTTGATGGCTGGAT-3'
Abcg5	5'-TCAATGAGTTTTACGGCCTGAA-3'	5'-GCACATCGGGTGATTTAGCA-3'
Abcg8	5'-TGCCCACCTTCCACATGTC-3'	5'-ATGAAGCCGGCAGTAAGGTAGA-3'
Abca1	5'-TCCTCATCCTCGTCATTCAAA-3'	5'-GGACTTGGTAGGACGGAACCT-3'
Cyp7a1	5'-AGCAACTAAACAACCTGCCAGTA-'3'	5'-GTCCGGATATTCAAGGATGCA-3'
Cyp8b1	5'-AGTGCCCTGAAACACACTCC-3'	5'-TCCTCCTGTACCACCCTGAG-3"
Shp	5'-CTCATGGCCTCTACCCTCAA-3'	5'-GGTCACCTCAGCAAAAGCAT-3'
ApoA-I	5'- TGTGTATGTGGATGCGGTCA-3'	5'- ATCCCAGAAGTCCCGAGTCA-3'
Abca1	5'- TCCTCATCCTCGTCATTCAAA-3'	5'- GGACTTGGTAGGACGGAACCT-3'
Abcg1	5'- CTCCGGCTTCCTCTTCTTCT-3'	5'- TACACGATGCTGCAGTAGGC-3'
18S	5'-TTGACTCAACACGGGAAACC-3'	5'-AGACAAATCGCTCCACCAAC-3'
